# An Online Tailored COVID-19 Vaccination Decision Aid for Dutch Citizens: Development, Dissemination, and Use

**DOI:** 10.2196/56390

**Published:** 2024-10-30

**Authors:** Katharina Preuhs, Daphne Bussink-Voorend, Hilde M van Keulen, Ilona Wildeman, Jeannine Hautvast, Marlies Hulscher, Pepijn van Empelen

**Affiliations:** 1 Netherlands Organization for Applied Scientific Research (TNO) Expertise Group Child Health Leiden Netherlands; 2 Radboud University Medical Center Radboud Institute for Health Sciences, Primary and Community Care Nijmegen Netherlands; 3 Radboud University Medical Center IQ Health Science Department (IQ Health) Nijmegen Netherlands

**Keywords:** COVID-19, COVID-19 vaccination, informed decision-making, user-centered design, low literacy, eHealth, tailored decision aid

## Abstract

**Background:**

Since December 2019, COVID-19 led to a pandemic causing many hospitalizations and deaths. Vaccinations were developed and introduced to control viral transmission. In the Dutch context, the decision to accept vaccination is not mandatory. An informed decision is based on sufficient and reliable information, in line with one’s attitudes and values, and with consideration of pros and cons. To support people in informed decision-making, we developed an online COVID-19 vaccination decision aid (DA).

**Objective:**

This article aims to describe the development, dissemination, and use of the DA.

**Methods:**

Building on a previously developed DA, the COVID-19 vaccination DA was developed in 3 phases following a user-centered design approach: (1) definition phase, (2) concept testing, and (3) prototype testing. End users, individuals with low literacy, and experts (with relevant expertise on medical, behavioral, and low literacy aspects) were involved in the iterative development, design, and testing, with their feedback forming the basis for adaptations to the DA.

**Results:**

The DA was developed within 14 weeks. The DA consists of 3 modules, namely, Provide Information, Support Decision-Making, and Facilitate Actions Following a Decision. These modules are translated into various information tiles and diverse functionalities such as a knowledge test, a value clarification tool using a decisional balance, and a communication tool. The DA was disseminated for use in May 2021. Users varied greatly regarding age, gender, and location in the Netherlands.

**Conclusions:**

This paper elaborates on the development of the COVID-19 vaccination DA in a brief period and its dissemination for use among Dutch adults in the Netherlands. The evaluation of use showed that we were able to reach a large proportion and variety of people throughout the Netherlands.

## Introduction

Since the identification of the first cases in 2019, COVID-19 has led to a pandemic impacting societies on a large scale. Vaccines became available in the Netherlands from January 2021 onward. Vaccination was voluntary, free, and introduced incrementally, starting with senior citizens and the most vulnerable, followed by adults, adolescents, and children. Those eligible for vaccination received an invitation from the National Institute for Public Health and the Environment, along with instructions to make an appointment at a regional vaccination center. In early 2021, there was significant hesitation in the population regarding the COVID-19 vaccines. Monitoring studies indicated that, particularly among individuals over 18 years of age, a considerable number of people were hesitant about getting vaccinated [1].

In the Dutch context, individuals are free to choose whether to be vaccinated. As this decision can impact health at both the individual and population levels, it is important that it is made in an informed and deliberate manner. A decision is considered informed when it is based on sufficient, reliable, and evidence-based knowledge, and when it aligns with people’s attitudes [2,3]. Additionally, a deliberate decision involves considering the advantages and disadvantages of vaccine acceptance or refusal [4,5]. Informed and deliberate decision-making reduces the likelihood of experiencing regret and decisional conflict, making individuals less vulnerable to misinformation and improving satisfaction with the decision-making process (eg, [6,7]). To make an informed and deliberate decision, people must understand all the necessary information involved in the decision-making process. Yet, 25% of the Dutch population—equating to 2.5 million citizens over the age of 16 years—have low health literacy. This means they face difficulties in finding, understanding, and applying information about illness and health, including following medical advice [8,9]. Low health literacy is more prevalent among the less educated, individuals with poorer perceived health [9], and those with a migrant background [8]. There is a clear correlation between limited health literacy and poorer health outcomes [8,9]. Decision aids (DAs) may help individuals with low health literacy make well-informed and deliberate decisions.

DAs assist users by breaking down the decision-making task into smaller steps, reducing the cognitive effort required to complete it [10]. Several reviews and meta-analyses have demonstrated that DAs are effective tools for supporting informed and deliberate decision-making, as they enhance knowledge, reduce decisional conflict, and increase satisfaction with the decision-making process (eg, [7,11]). This also applies to vaccination DAs [12]. In the Netherlands, online DAs have been developed for human papillomavirus vaccination and maternal pertussis vaccination. These aids provide information about infectious diseases and vaccination, allow users to weigh the pros and cons, and encourage reflection on important values related to their vaccination choices. Both DAs improved informed decision-making and reduced decisional conflict [13,14]. These resources served as a foundation for the development and dissemination of an online COVID-19 vaccination DA in the Netherlands.

The introduction of COVID-19 vaccines occurred rapidly, creating an urgent need for the swift development and dissemination of a DA to support informed and deliberate decision-making. We aimed to create a DA for individuals hesitant about vaccination by providing sufficient and reliable information, facilitating the decision-making process, and assisting users in making an appointment once they choose to get vaccinated. This study aims to provide a detailed description of the development, dissemination, and use of the COVID-19 vaccination DA in the Netherlands.

## Methods

### Principles for the Design of the COVID-19 Vaccination Decision Aid

The development of the DA was guided by the International Patient Decision Aid Standards (IPDAS) criteria whenever possible and applicable [15]. As the structure of the DA was based on earlier research [16,17], the overall framework and functionalities were already established and served as a blueprint for its development. Although this blueprint was generic and could be adopted directly, the content needed to be adapted for COVID-19 vaccination. For this, we utilized various sources, including information to address frequently asked questions (FAQs) about COVID-19 vaccines gathered from call centers of regional public health services and interviews conducted by the National Institute for Public Health and the Environment. We also incorporated input from experts and relevant literature, such as research on doubts and concerns about COVID-19 vaccination within the Dutch population [18]. All information in the DA was presented at a B1 language level to ensure accessibility for individuals with lower health literacy. The DA was developed as a mobile-first web app; earlier research indicated that most adults in the Netherlands use their mobile phones for internet access (82% in 2019) and search online for health information (69%) [19].

The adaptation to COVID-19 vaccination was carried out iteratively, applying user-centered design principles, which involved users in the development process [20] to enhance user engagement and the usability of the tool [19,21]. In accordance with the recommendations of the IPDAS collaboration, both users and experts participated in this process [22]. The DA was developed through the following phases: (1) definition phase, (2) concept testing phase, and (3) prototype testing phase. The different phases are described in more detail in Table 1 and in the subsequent subheadings.

**Table 1:** Overview of the iterative development approach and sample characteristics.

Phase	Goal	Means	Sample
Definition phase	Definition of the behavioral problem and program objectives, translation of evidence-based methods into behavioral strategies, and content-wise adaptation of the DA^a^ to a concept DA.	Literature study, interviews, call center input, expert input	Experts on vaccination, communication, or behavior change (n=14)
Concept testing phase	Testing of the basic concept to evaluate acceptance and usability, resulting in the development of a prototype.	Input via e-mails and meeting notes Online survey and online discussion	Experts regarding vaccination (n=7) Respondents (n=51) of a Dutch community panel
Prototype testing phase (including low literacy testing)	Validation of content and refinement and usability (and acceptance) testing to ensure fit among users including those with lower (health) literacy.	Expert review Interviews	Independent experts regarding vaccination (n=7); expert on low literacy (n=1) Individuals with low literacy (n=2)

^a^DA: decision aid.

The overarching theoretical method for developing the DA is tailoring, a communication strategy that adapts feedback to individual needs [23]. Tailored interventions have been shown to be more effective than generic interventions in changing behavior [24,25]. Additionally, tailoring enhances exposure, information processing, appreciation, and perceived personal relevance (eg, [26,27]). It also aligns well with one of the main principles of informed decision-making, which is to reduce cognitive effort [10]. Intervention mapping was used to systematically develop a tailored DA based on theory and evidence [28].

### Ethical Considerations

Both experts and users participated in the testing rounds of phases 2 and 3. Experts were selected based on their expertise in vaccination, communication, behavior change, and low literacy. Users were represented by a community panel, and we deliberately included individuals with low literacy skills to enhance the accessibility of the DA for this specific group. Users were recruited through “Foundation ABC,” which represents the interests of people with low literacy. The feedback provided by participants was analyzed anonymously to ensure privacy. Community panel members and individuals with low literacy were informed about the study’s purpose and provided informed consent. They received a gift voucher for their participation, and their contact information was deleted afterward.

The study received ethical approval from the Netherlands Organization for Applied Scientific Research (TNO) Institutional Review Board for Human Research (reference number 2020–041).

### Blueprint of the COVID-19 Vaccination Decision Aid

#### Overview

**Figure 1:**
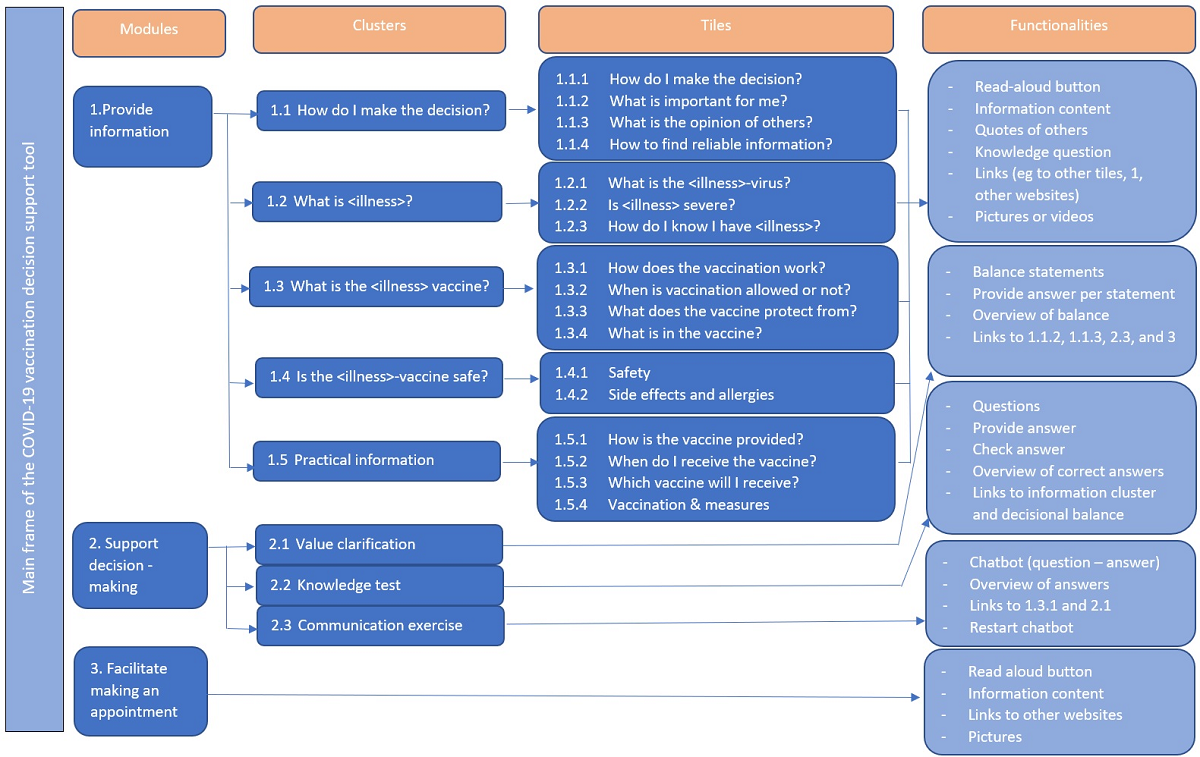
Blueprint for the COVID-19 vaccination decision support tool.

The DA blueprint is presented in Figure 1 and shows a framework consisting of modules, clusters, tiles, and functionalities. The *modules* represent the objectives to provide information, support decision-making, and facilitate making an appointment once a user decides to get vaccinated. The *clusters* represent the main questions or components within each module. For example, module 1, “Provide Information,” consists of 5 clusters, each addressing a key question about the vaccine: “How do I make a decision about vaccination?” (1.1), “What is the illness?” (1.2), “What is the vaccine?” (1.3), “Is the vaccine safe?” (1.4), and “Practical information” (1.5). Each cluster is further subdivided into separate tiles linked to pages containing answers and additional information. For instance, cluster 1.2, “What is the illness?,” consists of 3 tiles with general information about the virus (tile 1.2.1), its severity (tile 1.2.2), and how people can know if they have the virus (tile 1.2.3). The *functionalities* describe how the information is presented, using various formats such as text blocks, quotes, questions, statements, videos, and links to other websites. Additional functionalities include a knowledge test, a chatbot to help prepare for conversations, and value clarification through an unbiased decisional balance exercise (2.1). This exercise consists of statements (eg, “I want to protect elderly and vulnerable people”) where users indicate the importance of each statement and whether it influences their decision to accept or refuse vaccination. Afterward, users receive an overview of the statements important to their decision, along with links for further information. In this way, the decisional balance exercise provides users with information tailored to their individual preferences.

#### Definition Phase

In the definition phase, we identified the behavioral problem and established program objectives to address it (step 1 of intervention mapping) [28]. Based on the literature, expert input, and FAQs, we inferred that people found it challenging to make a decision about COVID-19 vaccination and often hesitated. Thus, the behavioral problem was defined as “people who hesitate perceive the decision on whether or not to be vaccinated against COVID-19 as difficult and therefore find it difficult to make an informed and deliberate choice.” Objectives were derived from previous research [16,17]. Our program objectives included providing information, supporting decision-making, and facilitating actions following a decision. The importance of these objectives in the context of COVID-19 vaccination has been underscored by the National Institute of Public Health and the Environment [29].

Subsequently, we gathered information on potential determinants of the behavioral problem, as well as on the information needs representing topics that could be addressed by the DA. We utilized relevant literature, expert opinions, and an overview of FAQs on COVID-19 vaccination collected by national and regional public health offices. Common considerations for informed and deliberate decision-making included short- and long-term side effects (ie, beliefs regarding the safety of the vaccine), protecting others through vaccination, and the desire to overcome the crisis by being vaccinated (ie, outcome expectancies and beliefs about effectiveness). Additionally, factors such as trust in science and institutions, as well as the desire for personal protection through vaccination, were significant [30]. Other considerations included prior vaccination behavior, trust in the government and policies related to COVID-19 measures and vaccination (ie, trust and emotions/affect), perceived severity of COVID-19 (ie, risk perception), and perceived vulnerability of oneself and others [18,30]. Furthermore, hesitant individuals expressed a need for reliable information regarding the content, safety, and effectiveness of the vaccine; the experiences of others with the vaccine; and the consequences of their decision for themselves and others [18,30]. Altogether, a wide array of considerations is relevant for informed and deliberate decision-making about COVID-19 vaccination. These include knowledge, risk perception, beliefs about vaccine efficacy, side effects, social norms, and self-efficacy [1,31-35].

As the next step in intervention mapping, we selected the most important and changeable determinants that could potentially influence the objectives. The 14 selected determinants included knowledge, attitudes toward the COVID-19 vaccination, outcome expectancies, beliefs about safety, beliefs about effectiveness, risk perception, moral norms, trust, perceived control, descriptive norms, injunctive norms, social pressure, emotion/affect, and decisional uncertainty. Next, we selected theory- and evidence-based methods to address each determinant in the DA, drawing on earlier research by Eldredge et al [28] and Kok et al [36].

For example, to address the determinant of knowledge, we applied the method of *active learning* [37,38] by including a knowledge test module featuring 10 statements about COVID-19 and its vaccination, as well as knowledge questions on most information pages. Additionally, we used the method of *chunking* [39] to enhance knowledge retention by grouping the information module into different tiles and using subheadings to divide each content page into smaller text blocks. Furthermore, to address users’ attitudes toward the COVID-19 vaccination and their decisional certainty, we selected the method of providing *feedback on benefits and barriers* [40]. This was operationalized through a value clarification exercise (from the “support decision-making” cluster), where users were encouraged to reflect on how various reasons for or against vaccination (eg, concerns about potential side effects or the desire to protect others) might influence their choice to get vaccinated or not. After deciding whether these reasons applied to them, users received feedback in the form of a comprehensive summary of their personal decisional balance, enabling them to draw their own conclusions. Another example is the determinant “perceived control,” which was addressed using methods that *provided information about others’ approval* [41,42] and *resistance to social pressure* [43]. This was implemented in the “communication exercise” cluster, where users could prepare for conversations with significant others about the COVID-19 vaccination and their decision, as well as seek opinions, support, understanding, or help from friends, family, or partners.

As illustrated by these examples, we used theory-based behavior change methods to address evidence-based determinants in each component of the DA and made corresponding content adaptations to the blueprint. This process resulted in a functional concept version of the DA, which was ready for use as a web application. From this point, we proceeded to the testing phases.

#### Concept Testing Phase

The concept version was tested by a group of experts in vaccination (n=7; 3 males and 4 females) and 51 respondents from a Dutch community panel, “Nederland denkt mee Community”.

The experts included scientists with expertise in vaccination, communication, or behavior change. They provided written feedback on the information presented in the DA, the balance between written and visual content, the navigation through the tool, and users’ expectations. Additionally, the concept version of the DA was evaluated by the community panel through an online survey and discussion. Overall, the panel evaluated the DA as clear, understandable, trustworthy, neutral, and informative. Panelists who intended to be vaccinated or had already received the vaccine indicated that using the tool aligned with their decision. However, those who were critical of vaccination felt the information was too superficial to aid their decision-making. Most participants regarded Radboudumc, an academic hospital and source of the information, as an independent party, which contributed to the trustworthiness of the DA.

Both the expert group and the panel expressed overall positivity about the basic concept of the DA and supported further development and adaptation of the initial version. Feedback from both groups was evaluated and incorporated into the prototype of the DA where possible and applicable. Illustrative examples of specific suggestions, feedback, and the actions taken are provided in Table 2.

**Table 2:** Examples of feedback from participants on the concept and prototype and consequential adaptations for improvement.

Phase: input source and feedback			Adaptations
**Concept testing: experts regarding vaccination uptake, experts on behavior change, and respondents of a community panel**			
	*It could be made more clear that the decision aid does not give choice advice, but that users must make their own choice and that the decision aid supports them in making that decision.*	The sentences explaining and introducing the “Support Decision-Making” module were rephrased.	
	*The testimonials about experiences of others is anecdotical and one-sided. Instead more factual information is preferable.*	Testimonials of health care professionals were added and supplemented with experiences from a professional point of view.	
**Prototype testing: experts regarding vaccination uptake**			
	*The information and recommendation on vaccination during pregnancy has changed.*	The information was adjusted in line with recent insights and advice.	
	*The text blocks contain too much information, this diminishes readability.*	The amount of information was reviewed, lengthy text blocks were shortened, and weblinks to additional information were added.	
**Prototype testing: individuals with low literacy and low-literacy expert**			
	*Include a read-aloud function*	A read-aloud function was installed and a button appeared on each page of the decision aid.	
	*Readability could be improved. Review the Corona glossary, a list of easy to understand words used to explain COVID-19 related medical matters.*	The glossary was reviewed and wording was adjusted accordingly, where possible. Where we could not avoid the use of long or complex words, we added hyphens (eg, corona-vaccination) to improve readability.	

#### Prototype Testing Phase

In the final phase, the prototype of the DA was retested by the same experts in behavior change (n=7). To maximize usability and acceptability among individuals with low literacy skills, the prototype was also tested by 2 individuals with low literacy and a low-literacy expert. The experts reviewed the prototype and provided written feedback. Overall, they felt that the DA supported users’ decision-making in a neutral manner. Additionally, they suggested more detailed adjustments to the information on certain topics and the wording of statements in the value clarification exercise.

The individuals with low literacy and the low-literacy expert reviewed the DA and were interviewed afterward. The low-literacy expert highlighted that the value clarification exercise (2.1) and knowledge test components (2.2) were particularly beneficial for individuals with low literacy skills. Consequently, we decided to give these components a more prominent position on the home page of the DA to enhance their visibility. The 2 individuals with low literacy suggested adjustments to enhance readability and recommended the addition of a read-aloud feature. Table 2 provides detailed and illustrative examples of feedback from all sources, along with the actions taken in response. In conclusion, this round of testing led to modifications that improved the accessibility of the DA for people with low literacy skills, optimizing the final version for dissemination.

#### Dissemination and Use of the COVID-19 Vaccination Decision Aid

The DA was disseminated through online news articles, social media platforms, and the website of the National Institute for Public Health and the Environment. To specifically increase its reach among the low-literacy target group, printed leaflets containing information about the DA were distributed at general practitioner offices and pharmacies.

Usage was assessed through Google Analytics (Google LLC/Alphabet Inc.), covering the period from the launch on May 18, 2021, to February 1, 2022. This included data on the number of users, their characteristics, and the time spent on various components of the DA.

## Results

### The COVID-19 Vaccination Decision Aid

The DA was developed over 14 weeks, from February to May 2021, and went live on May 18, 2021. This timeline coincided with the invitations for COVID-19 vaccinations for individuals aged 18 and older. Special attention was given to specific questions that individuals might have, such as those regarding pregnancy or concerns for particularly vulnerable groups (eg, older adults or individuals with chronic diseases). From that point onward, the DA was made accessible to the general population as a mobile-first web app.

### Navigation

Users arrive at the home page of the DA, where they are directed to the cluster supporting decision-making. They will see 4 tiles: “How do I make the choice?,” “What is important to me?,” “What do others think?,” and “How does the vaccination work?.” These tiles represent the most essential elements of the DA.

From the home page, users can also navigate to the “Provide Information” module. This module is organized into different clusters and tiles, following the blueprint structure. Each information tile features a heading that displays a specific question or topic, accompanied by a matching icon. When a tile is selected, users are directed to a content page dedicated to that particular topic. Each information page follows a similar structure, featuring short paragraphs with clear headers that introduce the content. Links to more in-depth information are provided for those interested, in line with the IPDAS guidelines [44]. Some pages also include videos, images, testimonials, and short quotes relevant to the topic. This variety in formats enhances accessibility for a wide range of users [45]. To improve readability for individuals with low health literacy, the pages have high visual contrast, concise text blocks written at a B1 level, and often use bullet points. Additionally, a read-aloud function is available.

Throughout the various components of the DA, a menu at the bottom of the page remains accessible, allowing users to navigate to the home page, the “Provide Information” module, the “Support Decision-Making” module, or the FAQs. The FAQs contain practical information on how to receive the vaccination and provide referrals to other trusted sources, such as the Health Council, the Pharmacovigilance Centre, and the Ministry of Health. This module was included to meet users’ needs for relevant practical information to support their decision-making.

Since the launch of the DA in May 2021, new information regarding COVID-19, such as the emergence of new variants, the extension of vaccination advice to children, and the introduction of boosters, necessitated regular updates to the DA’s content. These developments required the continuous involvement of members of the development team to ensure the information remained accurate and up to date.

### The Support Decision-Making Module

**Figure 2:**
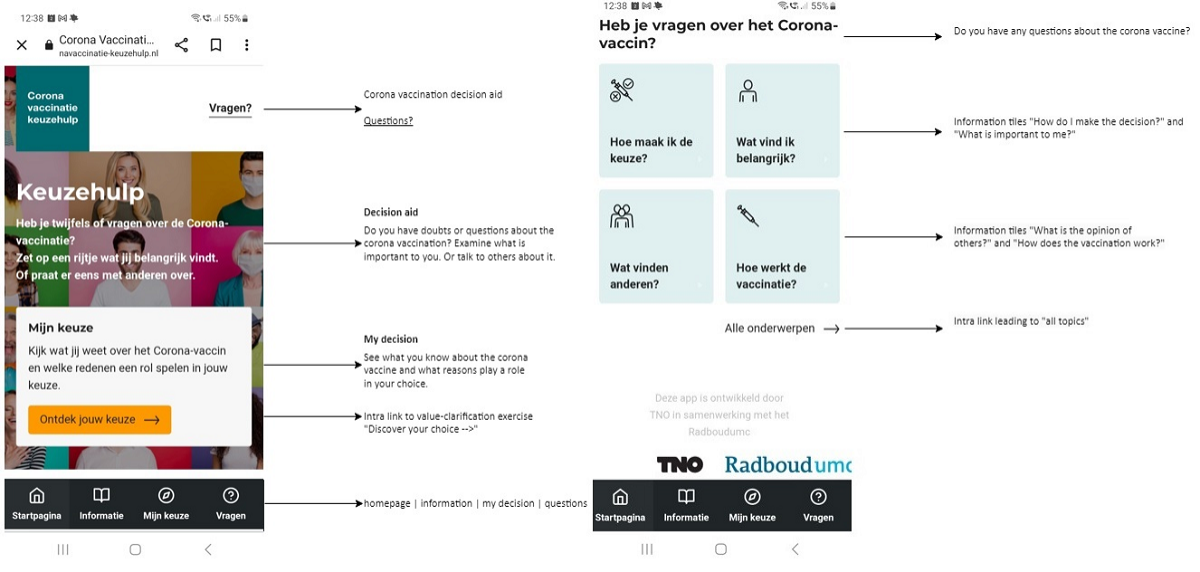
Home screen of the COVID-19 vaccination decision aid and content elements.

The most prominent area of the home screen (Figure 2) features the “Support Decision-Making” module, encouraging users with the shortcut: “See what you know about the corona vaccine and what factors influence your decision.” Additionally, users can directly access information tiles such as “How do I make the decision?,” “What is important to me?,” “What do others think?,” and “How does the vaccination work?,” followed by a link to view “all topics.”

**Figure 3:**
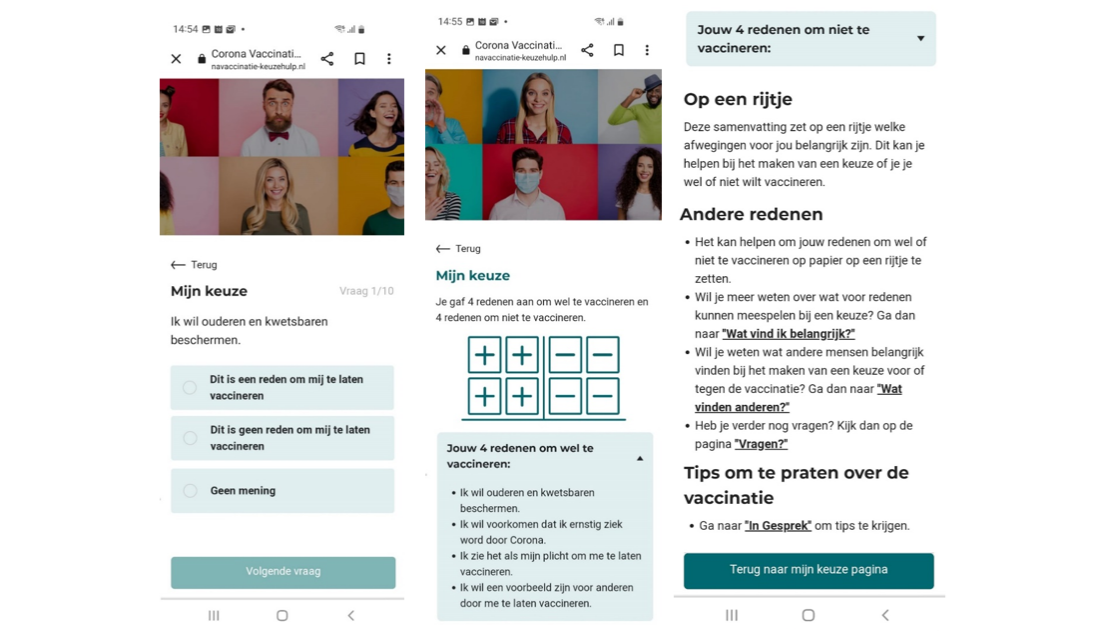
Value clarification exercise.

These consist of 3 clusters: (1) value clarification (Figure 3), (2) a knowledge test, and (3) a communication exercise.

In the decisional balance cluster (Figure 3), users are presented with 10 statements (e.g., “I want to protect elderly and vulnerable people” or “I am seriously worried about potential side effects”). They can respond by indicating whether each statement serves as a reason to accept or refuse vaccination, or if it does not affect their decision. The statements address personal values related to medical information about the vaccination and COVID-19, the pandemic, and the role of vaccines. After completing the value clarification, users receive feedback in the form of a comprehensive summary of their personal considerations, helping support their decision-making process. This feedback is supplemented with suggestions to discuss these considerations with friends, family, or their partner, as well as links to relevant tiles within the DA. Importantly, users do not receive explicit advice for or against vaccination, allowing them to make their own informed decisions based on their values and preferences.

The knowledge test comprises 10 statements addressing common misconceptions, such as those related to infertility, vaccine safety, and vaccination during pregnancy. Users can respond with “true,” “false,” or “I don’t know” and receive immediate feedback after each response. Upon completing the test, they are provided with an overview of the number of correct answers, helping to reinforce accurate information and dispel myths.

The communication exercise is designed to help users prepare for conversations with others about their vaccination decisions. It prompts users to reflect on the goal of the conversation, whether it is to seek support, understand the other’s opinion, gain an understanding of their own decision, or try to convince the other person. Users are also guided to articulate their own viewpoint on vaccination, explain their reasons, express how they feel, and state what they need from the other person. The exercise concludes by providing users with a summary of their responses, helping them to engage more effectively in these discussions.

Links to each of the 3 clusters are provided throughout the “Support Decision-Making” module.

### Dissemination and Use of the COVID-19 Vaccination Decision Aid

Website statistics showed that the DA was used by 57,567 individuals between May 18, 2021, and February 1, 2022. Peak usage occurred during key periods, including the launch of the adolescent vaccination campaign (June to July 2021), the introduction of booster vaccines (November 2021), and the recommendation for children aged 5-11 years to be vaccinated (January 2022). The age distribution of users varied: of the 57,567 users, 12,434 (21.60%) were aged 65 years and older; 12,204 (21.20%) were between 55 and 64 years; 11,053 (19.20%) were between 45 and 54 years; 8462 (14.70%) were aged 35-44 years; 9787 (17%) were between 25 and 34 years; and 3627 (6.30%) were aged 18-24 years, according to Google Analytics. The user demographics included both males (35,289/57,567, 61.30%) and females (22,278/57,567, 38.70%).

**Figure 4:**
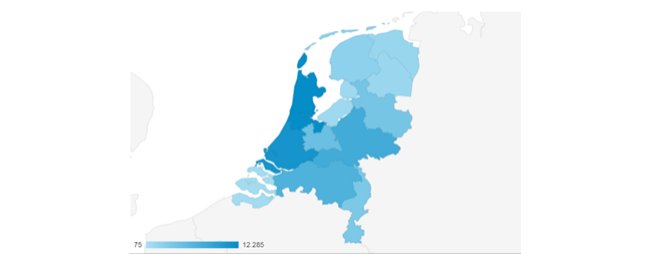
Distribution of the use of the COVID-19 vaccination decision aid in the Netherlands.

The DA was disseminated through various municipal health services and local newspapers. Most users (36,152/57,567, 62.80%) accessed the DA from their mobile phones, while others used their desktops (19,343/57,567, 33.60%) or tablets (2072/57,567, 3.60%). On average, users spent 3 minutes and 31 seconds on the DA. Figure 4 displays the distribution of users across the Netherlands, with a higher number of users in the provinces of North and South Holland. Table 3 outlines the number of page clicks among users of the DA.

**Table 3:** Number of clicks among users per page of the COVID-19 vaccination decision aid.

Module: cluster (decision aid page)	Clicks, n
Support Decision-Making	36,133
Support Decision-Making: Decisional balance (2.1)	35,947
Support Decision-Making: Knowledge test (2.2)	12,890
Provide Information: How do I make a decision? (1.1.1)	11,380
Provide Information: How does the vaccination work? (1.3.1)	5347
Support Decision-Making: Communication exercise (2.3)	3749
Provide Information: What is important for me? (1.1.2)	3642
Provide Information: Which vaccine will I receive? (1.5.3)	3226
Provide Information: Side effects and allergies (1.4.2)	2983
Provide Information: What is in the vaccine? (1.3.4)	2911
Provide Information: What is the opinion of others? (1.1.3)	2704
Provide Information: Vaccination and measures (1.5.4)	2350
Provide Information: Safety (1.4.1)	2166
Provide Information: How to find reliable information? (1.1.4)	1875
Provide Information: Is COVID-19 severe? (1.2.2)	1266
Provide Information: How is the vaccine provided? (1.5.1)	989
Provide Information: How do I know if I have COVID-19? (1.2.3)	783
Provide Information: What is the COVID-19 virus? (1.2.1)	524
Readspeaker: Read aloud	439

### Use of the Support Decision-Making Module

The tile “How do I make a decision” (1.1.1) received the most clicks (n=11,380) within the “Provide Information” module. Overall, the “Support Decision-Making” module, which includes 3 clusters—decisional balance (2.1), knowledge test (2.2), and communication exercise (2.3)—garnered the highest number of clicks (n=36,133). Within this module, the decisional balance cluster had 35,947 clicks, followed by the knowledge test with 12,890 clicks. The communication exercise was the least utilized cluster of the Support Decision-Making module.

Table 4 provides further details on the value clarification exercise (cluster 2.1). The most frequently reported reasons against vaccination included concerns about safety (22,287/30,670, 72.67%) and side effects (22,003/32,405, 67.90%), the belief that one’s healthy lifestyle is sufficient to protect against COVID-19 (15,796/31,565, 50.04%), and the inability to choose which vaccine to receive (14,803/29,853, 49.59%). Conversely, the primary reasons in favor of vaccination were to prevent serious illness (18,016/31,030, 58.06%) and to protect others, particularly vulnerable individuals (14,621/33,565, 43.56%). The most frequently reported reasons considered not important to the decision were perceiving vaccination as a duty (7672/30,426, 25.22%) and being an example (5527/30,042, 18.40%).

**Table 4:** Percentage of users who selected a reason in favor of vaccination, as not important, or unfavorable of vaccination within the decisional balance cluster (N=33,565).

Reason	In favor of vaccination, n (%)	Against vaccination, n (%)	Not important, n (%)
I am concerned about serious side effects (n=32,405)	2592 (8.00)	22,003 (67.90)	7810 (24.10)
I want to protect older and vulnerable people (n=33,565)	14,621 (43.56)	4085 (12.17)	14,859 (44.27)
I want to prevent myself from getting seriously ill (n=31,030)	18,016 (58.06)	2737 (8.82)	10,277 (33.12)
I see it as my duty to be vaccinated (n=30,426)	7672 (25.22)	3104 (10.20)	19,650 (64.58)
I want to be an example by being vaccinated (n=30,042)	5527 (18.40)	5383 (17.92)	19,132 (63.68)
I think we will get out of the crisis by means of vaccinating (n=29,577)	11,610 (39.25)	5597 (18.92)	12,370 (41.82)
I am concerned about the safety of the vaccine (n=30,670)	2034 (6.63)	22,287 (72.67)	6349 (20.70)
I protect myself against COVID-19 in a natural way (n=30,201)	5656 (18.73)	11,814 (39.12)	12,731 (42.15)
My lifestyle is healthy enough to protect me against COVID-19 (n=31,565)	3805 (12.05)	15,796 (50.04)	11,964 (37.90)
I only want to be vaccinated if I can choose which vaccine I receive (n=29,853)	7935 (26.58)	14,803 (49.59)	7115 (23.83)

Table 5 displays the responses provided by users on the knowledge test items up until May 24, 2022. In most instances, the majority of users selected the correct answers. Most users understood that after receiving the vaccination, they were less likely to become ill, that they might experience side effects, that it is inadequate to vaccinate only older and vulnerable individuals, and that they cannot choose which vaccine to receive. Furthermore, most users understood that after receiving the vaccination, they were still required to maintain a distance of 1.5 m and that testing was still necessary. However, users demonstrated the least knowledge regarding vaccination during pregnancy, with half believing that they should postpone vaccination while pregnant. In addition, users were less clear that the same requirements for development and authorization applied to the COVID-19 vaccine as for any other vaccine developed in Europe.

**Table 5:** Percentage of users who answered true, false, or I don’t know on the knowledge test items (N=12,890).

Reason	True, n (%)	False, n (%)	I don’t know, n (%)
If I have had the vaccination I am less likely to get sick.	7784/9730 (80.00^a^)	1602/9730 (16.46)	344/9730 (3.54)
If I have had the vaccination I don’t need to keep 1.5 m distance anymore.	580/7448 (7.79)	6726/7448 (90.31^a^)	142/7448 (1.91)
If I have had the vaccination I don’t need to get tested anymore.	96/1881 (5.10)	1758/1881 (93.46^a^)	27/1881 (1.44)
When pregnant I can better postpone the vaccination.	4377/10,568 (41.42)	5228/10,568 (49.47^a^)	963/10,568 (9.11)
It is sufficient to vaccinate only older and vulnerable people.	1710/8837 (19.35)	6727/8837 (76.12^a^)	400/8837 (4.53)
The COVID-19 vaccination can lead to infertility.	1271/8621 (14.74)	5844/8621 (67.79^a^)	1506/8621 (17.47)
The vaccination also protects against the British variant of the COVID-19 virus.	5627/7185 (78.32^a^)	941/7185 (13.10)	617/7185 (8.59)
The vaccination also protects against the delta variant of the COVID-19 virus.	1197/1586 (75.47^a^)	290/1586 (18.28)	99/1586 (6.24)
The vaccination also protects against the Omicron variant of the COVID-19 virus.	190/245 (77.55^a^)	42/245 (17.14)	13/245 (5.31)
The vaccine is being assessed in the Netherlands and Europe.	6799/8380 (81.13^a^)	1098/8380 (13.10)	483/8380 (5.76)
Everyone can choose the Janssen vaccine.	454/1864 (24.36)	1211/1864 (64.97^a^)	199/1864 (10.68)
I can choose myself which vaccine I will receive.	1036/7325 (14.14)	6070/7325 (82.87^a^)	219/7325 (2.99)
You can get muscle aches and headaches from the vaccination.	9719/9948 (97.70^a^)	79/9948 (0.79)	150/9948 (1.51)
Different requirements (than for other vaccines) have been handled for the COVID-19 vaccine.	4206/11,419 (36.83)	6200/11,419 (54.30^a^)	1013/11,419 (8.87)

^a^Correct answer.

The communication exercise was the least utilized cluster within the Support Decision-Making module. In this exercise, users were prompted to consider what they would like to share with their communication partner. Users indicated their need to feel understood and receive support (eg, “It is important for me that you accept my choice. Even though you might not agree with me.”, “I need your support and help not to be persuaded/talked around by others”); provide their reasons for their decision (eg, “The development went too fast. The long-term side effects are still unknown. I always react heavily on medication”); share their doubts and fears (eg, “The vaccine I NEED to take only protects me for 60%”) and trying to understand what the opponent wants themselves, and why (eg, “How do you feel about it? Why aren’t you afraid or suspicious?”); and the influence of their own decision on the other (eg, “What do you think of me wanting to be vaccinated? Does this affect your own choice?”).

## Discussion

### Principal Findings

This article describes the development, dissemination, and use of a DA for individuals hesitant about the COVID-19 vaccination in the Dutch population. The DA was developed quickly, within a time frame of 14 weeks, by utilizing a blueprint based on previous, evidence-based, and effective DAs for vaccinations [13,14]. This provided a solid foundation and structure throughout the development process. The DA included modules for providing information, supporting decision-making, and facilitating appointment scheduling. The “Support Decision-Making” module was the most utilized, indicating that our efforts to help individuals make informed and deliberate choices about vaccination have been successful.

The average time users spent on the DA was 3.5 minutes, which aligns with evaluations of similar DAs [13,45]. Users most frequently navigated to the Support Decision-Making module, particularly the decisional balance and knowledge test clusters, as well as the information page titled “How do I make a decision.” The least used component was the communication exercise cluster. Compared with a DA on maternal pertussis vaccination [17], our results are similar in terms of usage for both the decisional balance cluster (most visited) and the communication exercise cluster (least visited). Additionally, both DAs demonstrate significant user engagement regarding information on vaccine side effects and safety. This aligns with our input for the DA, which indicated that these topics are relevant doubts and considerations contributing to vaccination hesitancy.

The knowledge test revealed that users had the least understanding of the need for COVID-19 vaccination during pregnancy and the requirements for vaccine development and authorization in Europe. Future communication about vaccination should consider these knowledge gaps.

In our DA, the most frequently selected reasons for accepting COVID-19 vaccination were to prevent illness and to protect others. Conversely, the most common reasons for refusing the vaccine were concerns about side effects and safety. This aligns with other research (eg, [46]) and highlights the importance of addressing these issues in future communication about vaccination.

By utilizing various dissemination channels (eg, general practitioners, the website of the National Institute for Public Health and the Environment) and methods (eg, links, leaflets), we reached a diverse group of 57,567 individuals across different genders, ages, and locations in the Netherlands. Peaks in DA usage (up to 3000 users per day) coincided with moments when specific age groups received invitations to get vaccinated, indicating that the relevant target audiences were effectively reached. As many studies do not report the populations they engage in, it is challenging to compare results. Therefore, documenting reach is essential in future studies.

In this paper, we described the iterative development process that involved experts in vaccination, communication, and behavior change, as well as end users, including individuals with low literacy skills. This co-design approach is crucial for enhancing the interest, attractiveness, understandability, and implementation of interventions [28]. Additionally, involving both experts and end users is essential for the development, reach, effectiveness, use, and acceptability of an intervention. As this aspect is often overlooked or underreported, it is considered a strength of our study [47].

### Limitations

A primary limitation of this study is the lack of an evaluation of the DA’s effectiveness through a randomized controlled trial, which was necessitated by the rapid development and urgent need for the DA. While the value clarification exercise identified important reasons that Dutch citizens may have for (not) wanting to get vaccinated, it remains unclear to what extent the DA influenced users’ opinions regarding their initial decisions. However, the foundation of our DA was based on previous evidence-based DAs that demonstrated positive effects on informed decision-making, helping users become more certain of their choices, as well as influencing determinants of vaccine acceptability and achieving adequate acceptability among the target group [13,14]. We observed that the DA was utilized by 57,567 individuals. While this represents a considerable reach, the Central Bureau of Statistics (CBS) [48] reports that 59% of the Dutch population was aged between 20 and 65 years in 2022, which translates to approximately 10.2 million Dutch citizens. In research conducted by RIVM [1], 1 in 5 surveyed individuals indicated hesitation regarding vaccination, translating to approximately 2 million people. Therefore, if active dissemination strategies had been implemented through a campaign, they could have led to even greater uptake among the Dutch population. This is evidenced by another initiative, a telephone hotline [49], which allowed Dutch citizens to ask questions related to COVID-19. The hotline was actively promoted through national newspapers, television, and government communications, leading to a larger uptake; it reached 200,000 people within 2 months. By contrast, the dissemination of the DA was primarily via municipal health services and local newspapers. While the daily number of hotline users (around 1000-1500) was comparable to the DA’s peak usage (1000-3000 users per day), the DA could have benefited from more active dissemination strategies to achieve a greater reach, similar to that of the hotline.

### Strengths

This study has several strengths. First, the involvement of experts and potential end users in the development of the DA is particularly noteworthy, especially given the brief time frame in which it was created. Additionally, the use of previously developed effective tools provided a solid blueprint for the rapid completion of the DA. The theory-informed approach to addressing determinants of vaccination decision-making also enhances the study’s robustness. Next, many DAs currently lack accessibility, as they do not meet the needs of individuals with low literacy skills [50]. In this study, we aimed to adhere to the principles of inclusive design by involving both an expert and users with (former) low literacy skills. The added value of the current DA, compared with standard communication about vaccination, lies in its interactive, tailored functionalities, and strategies, such as value clarification, a communication tool, and *chunking*. Another strength is the systematic development process using intervention mapping, which ensures a theory- and evidence-based approach [28]. Additionally, the underlying blueprint provides a template for efficient and high-quality development in response to other urgent health issues [51]. Finally, the DA was regularly updated during its dissemination and use, incorporating changes in policy and developments related to the virus and vaccination efforts as they became available. Our findings align with observations from others that suggest DAs significantly enhance patient empowerment, particularly in contexts where traditional shared decision-making was hindered by lockdowns. Our results contribute to the evidence that the rapid development and dissemination of online DAs can aid COVID-related decisions and may also apply to other urgent decision-making scenarios [51]. Such DAs may be even more crucial in contexts where the information provided is politicized rather than based on scientific facts, as was the case in some countries [52].

### Conclusions

Using evidence-based DAs as a foundation, we developed a COVID-19 vaccination DA in a short time frame and disseminated it for use among Dutch adults. The evaluation of its use indicated that we successfully reached a large and diverse segment of the population across the Netherlands.

## Abbreviations

DA: decision aid
FAQ: frequently asked question
IPDAS: International Patient Decision Aid Standards

## Data Availability

The data sets generated during or analyzed during this study are available from the corresponding author upon reasonable request.
